# Mpox multiprotein virus-like nanoparticle vaccine induces neutralizing and protective antibodies in mice and non-human primates

**DOI:** 10.1038/s41467-025-59826-8

**Published:** 2025-05-21

**Authors:** Ahmed A. Belghith, Catherine A. Cotter, Maxinne A. Ignacio, Patricia L. Earl, Rory A. Hills, Mark R. Howarth, Debra S. Yee, Jason M. Brenchley, Bernard Moss

**Affiliations:** 1 Laboratory of Viral Diseases, National Institute of Allergy and Infectious Diseases, National Institutes of Health, Bethesda, MD USA; 2 Department of Biochemistry, University of Oxford, Oxford, UK; 3 Department of Pharmacology, University of Cambridge, Cambridge, UK

**Keywords:** Preclinical research, Vaccines

## Abstract

The upsurge of mpox in Africa and the recent global outbreak have stimulated the development of new vaccines and therapeutics. We describe the construction of virus-like particle (VLP) vaccines in which modified M1, A35 and B6 proteins from monkeypox virus (MPXV) clade Ia are conjugated individually or together to a scaffold that accommodates up to 60 ligands using the SpyTag/SpyCatcher nanoparticle system. Immunisation of female mice with VLPs induces higher anti-MPXV and anti-vaccinia virus (VACV) neutralizing antibodies than their soluble protein (SP) counterparts or modified VACV Ankara (MVA). Vaccination with individual single protein VLPs provides partial protection against lethal respiratory infections with VACV or MPXV clade IIa, whereas combinations or a chimeric VLP with all three antigens provide complete protection that is superior to SPs. Additionally, the VLP vaccine reduces the replication and spread of the virus at intranasal and intrarectal sites of inoculation. VLPs induce higher neutralizing activity than the Jynneos vaccine in rhesus macaques, and the VLP-induced antiserum provides better protection against MPXV and VACV than the Jynneos-induced antiserum when passively transferred to female mice. These data demonstrate that an mpox VLP vaccine derived from three MPXV clade Ia proteins protects against clade IIa MPXV and VACV, indicating cross-reactivity for orthopoxviruses.

## Abstract (web summary)

The upsurge of mpox has stimulated the development of new vaccines and therapeutics. Here, the authors describe a VLP vaccine comprised of modified MPXV proteins M1, A35, and B6 and demonstrate induction of protective antibodies in mice and non-human primates.

## Introduction

The upsurge of human mpox in Africa and the recent global outbreak emphasize the need to better understand the disease and improve methods for its prevention and control^1^. Mpox clinically resembles smallpox but is less severe and less transmissible^2,3^. The human disease is caused by monkeypox virus (MPXV), a member of the *Orthopoxvirus* genus of the *Chordopoxvirinae* subfamily that includes variola virus, the causative agent of smallpox, as well as vaccinia virus (VACV) used for vaccines^4^. Mpox is a zoonosis believed to be transmitted from rodents to humans, but human-to-human spread occurs and has been increasing^5,6^. Two MPXV clades, each with two subclades, are distinguishable by genome sequencing and are associated with different endemic regions, symptoms, and transmission^7–9^. Subclade Ia, endemic in central Africa, particularly in the Democratic Republic of the Congo (DRC), causes the highest incidence of severe disease; children are most frequently infected, and human spread occurs within families^10,11^. Subclade Ib, recently identified in the DRC, exhibits sustained human transmission linked to sexual activity^12^. Subclade IIa appears to be restricted to animals in Ghana except for a self-limited human outbreak that occurred without mortality in the USA in 2003 due to the importation of infected rodents^7^. Subclade IIb, endemic in Nigeria, is responsible for the 2022 global outbreak in which there was sustained human transmission associated with sexual activity, and mild disease^13,14^. The nucleotide sequence difference between clade I and clade II genomes is ~4–5%, whereas the differences within the two subclade I strains and the two subclade II strains are much less. However, the genetic basis for differences in virulence and transmission is unknown^1^. Because the essential orthopoxvirus genes are highly conserved, attenuated strains of VACV can serve as vaccines for both mpox and smallpox^15^. Jynneos, comprised of the live replication defective modified VACV Ankara (MVA), was licensed for the prevention of smallpox and mpox based on animal experiments, as well as safety and immunogenicity in clinical trials^16^. The global outbreak of mpox made it possible to ascertain the effectiveness of Jynneos for MPXV subclade IIb in humans. One vaccination with Jynneos provided 36–75% effectiveness in preventing mpox, while two vaccinations with Jynneos were 66–89% effective^17–19^, though anti-MPXV neutralizing antibody was low and short-lived^20–23^. The incomplete protection and low neutralization of MPXV, together with the limited availability of the vaccine, indicate a need to develop enhanced mpox vaccines using the latest technologies. Thus, several papers have reported animal experiments with candidate mpox mRNA vaccines that produce secreted or cell membrane-associated antigens^24–30^. Here we describe the construction of virus-like particle (VLP) mpox vaccines and the protective immune responses elicited by them in mice and non-human primates (NHPs).

The enhanced immunogenicity achieved by presentation of a repetitive array of immunogens on VLPs was shown for hepatitis B^31^ and subsequently for human papillomavirus^32^, hepatitis E virus^33^, and other systems^34,35^. For the present study, we used the SpyTag/SpyCatcher nanoparticle (NP) system, which enables the covalent linkage of multiple copies of the same or different antigens to a scaffold in vitro and facilitates modular multivalent display on VLPs^36–38^. Candidate malaria^39^, multivalent influenza virus^40–42^ and multivalent coronavirus^43–47^ VLPs produced using this technology elicit strong immune responses.

A crucial question regarding recombinant vaccines, particularly for complex viruses, is the choice of immunogen(s). Orthopoxviruses exist as two infectious forms: the mature virion (MV) and the enveloped virion (EV), which have different outer proteins^4^. Previous studies with VACV soluble proteins (SPs) indicated that the best protection in animal models occurred with a combination of the MV L1 (OPG 95) protein required for cell entry, and either or both A33 (OPG 162) and B5 (OPG 190) EV proteins required for virus spread^48^. Following this strategy, we constructed VLPs containing the individual MPXV clade Ia homologs of L1, A33, and B5, as well as a chimeric VLP containing all three proteins. VLPs inoculated intramuscularly (IM) with adjuvant induced higher VACV and MPXV neutralizing antibodies than the SPs or MVA and reduced virus replication and spread following potentially lethal intranasal (IN) and sublethal intrarectal (IR) infections of mice. Furthermore, NHPs immunized subcutaneously with VLPs developed neutralizing antibodies that passively protected mice against VACV and MPXV subclade IIa infections. These data suggest that VLPs constructed with subclade Ia MPXV M1, A35, and B6 have the potential to serve as alternatives to live attenuated VACV for mpox vaccination.

## Results

### Construction and characterization of MPXV protein VLPs

**Fig. 1:**
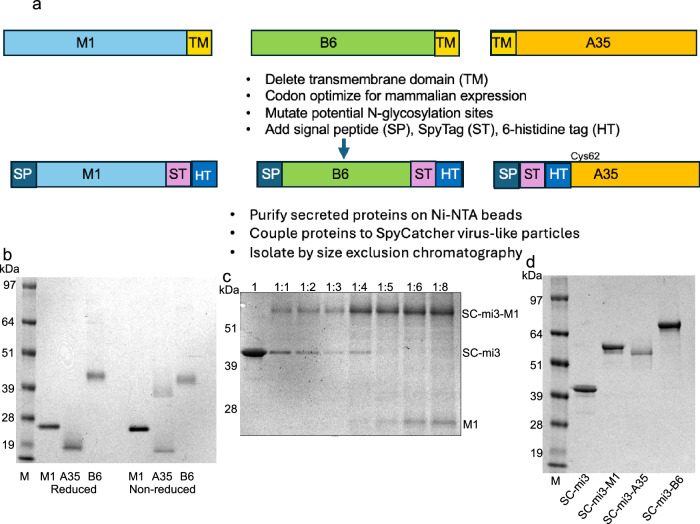
Construction of MPXV protein VLPs. **a** MPXV clade Ia M1, B6, and A35 proteins were modified for secretion and conjugated to a SpyCatcher scaffold (SC-mi3) with 60 attachment sites. **b** Secreted M1, A35, and B6 proteins fused to SpyTag were purified from the medium of transfected cells by Ni-NTA chromatography. Purified proteins were treated with SDS with (left) or without (right) a reducing agent and analyzed by SDS polyacrylamide gel electrophoresis. A Western blot probed with an antibody to histidine tag, representative of five independent purification experiments, is shown. The left lane labeled M shows marker proteins with their mass indicated in kDa. **c** Conjugation of M1 protein to SC-mi3. Purified SpyTagged M1 was conjugated to 2 µM SC-mi3 at indicated ratios and analyzed by SDS gel electrophoresis. A Coomassie blue-stained gel is shown. Marker proteins (M) at left, ratios of SC-mi3 to M1 at top, positions of SC-mi3-M1 conjugate, SC-mi3, and M1 on right. Similar conjugation results with 5:1 ratios were obtained in five separate experiments with M1, A35 and B6. **d** Coomassie blue-stained SDS gel analysis of SC-mi3 conjugates derived from purified VLPs used for immunization of mice. Similar results were obtained for five separate purifications.

We chose subclade Ia MPXV proteins because the virus causes severe disease, is increasing in incidence, has the potential for global spread, and has been well studied in animal models. M1, A35 and B6 of MPXV-ZAI-1979-005 (L1, A33 and B5 VACV homologs, respectively) were modified for the construction of the VLPs as diagrammed in Fig. 1a. The open reading frame (ORF) sequences were codon-optimized for expression in mammalian cells, the transmembrane (TM) domains deleted, and signal peptides added to enable secretion to facilitate purification. In contrast to A35 and B6, M1 does not traffic through the endoplasmic reticulum during a virus infection, and therefore, the putative *N*-glycosylation sites of M1 were mutated to prevent such unnatural modifications. A six-histidine tag and a SpyTag were added to each ORF to enable purification and coupling to the SpyCatcher003-mi3 (referred to as SC-mi3) scaffold, respectively. Each modified MPXV ORF was inserted into a plasmid mammalian expression vector and transfected into cells. After incubation, the clarified medium was incubated with Ni-NTA beads, which were then washed, and the bound proteins eluted with imidazole, coupled to SC-mi3, and further purified by size exclusion chromatography. SC-mi3 forms stable 60-subunit nanocages, allowing multimerization of antigens through spontaneous isopeptide bond formation in vitro between SpyTag and the SC-mi3 moiety^49^. We constructed monovalent VLPs containing multiple copies of M1, A35, or B6, as well as a chimeric multivalent VLP containing all three antigens.

The purified proteins, prior to coupling, migrated on SDS-polyacrylamide gel electrophoresis as single major bands of the expected mass under reducing conditions (Fig. 1b). A slowly migrating A35 band was detected under non-reducing conditions because Cys62 adjacent to the TM domain (Fig. 1a) was preserved so that a disulfide bond would be formed to stabilize native homodimers, which could double the amount of A35 in VLPs and possibly enhance immunogenicity. Based on an initial experiment with M1, a 1:5 molar ratio of SC-mi3 monomer to MPXV protein was selected for efficient conjugation (Fig. 1c). The VLPs were purified from unconjugated protein by size exclusion chromatography. Polyacrylamide gel analysis of SDS-dissociated purified VLPs to be used for immunizations demonstrated major SC-mi3-conjugated proteins of expected size (Fig. 1d).

### Immunogenicity of MPXV VLPs and protection in a lethal VACV challenge model

**Fig. 2:**
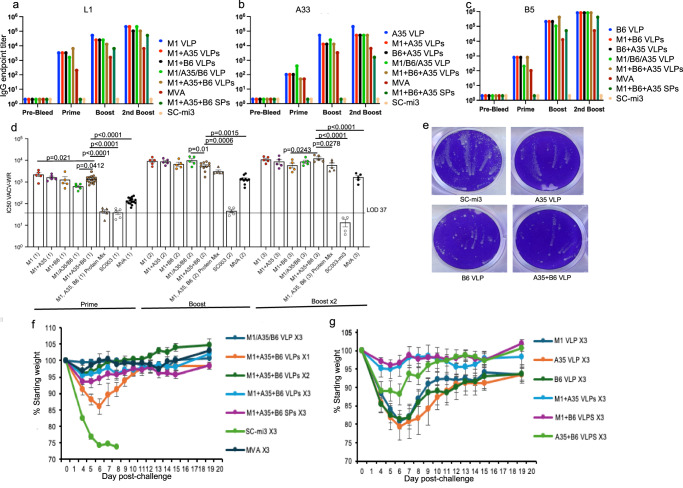
Vaccine-induced antibodies and protection against a lethal VACV challenge. **a**–**c** Binding antibodies detected by ELISA. BALB/c mice (*n* = 5 per group) were immunized IM three times at 3-week intervals with 2 µg of individual VLPs or combinations (+) containing 2 µg of each VLP, 6 µg of chimeric VLP (M1/A35/B6), 2 µg of unconjugated SC-mi3; 2 µg of each of the three SPs, or 10^7^ PFU of MVA. Sera from each group were pooled and endpoint binding titers to VACV determined in duplicate for L1, the homolog of M1 (**a**), A33, the homolog of A35 (**b**), and B5, the homolog of B6 (**c**). **d** Anti-VACV neutralization titers of serum from individual mice were determined in duplicate and plotted as NT50 geometric mean. Error bars—SEM, LOD limit of detection. Neutralization titers elicited by M1 VLPs alone or in combination with other VLPs were similar, and significances determined by one-way ANOVA with multiple comparisons test are shown only for the three VLP combinations relative to unconjugated SC-mi3, SPs, and MVA. **e** Inhibition of VACV spread by pooled A35 VLP, B6 VLP, A35 + B6 VLPs or SC-mi3 immune sera added 1 h after infection of cells and stained with crystal violet two days later. **f** Weight loss and survival of BALB/c mice (*n* = 5 per group) challenged IN with 10^6^ PFU of VACV WR at 3-weeks after 1 (X1), 2 (X2) or 3 (X3) immunizations with chimeric VLP, a combination of the three monovalent VLPs, a combination of the three SPs, SC-mi3 or MVA. Bars represent SD. **g** As in (**f**), except the animals were immunized three times with individual monovalent VLPs or combinations of two individual monovalent VLPs. Source data are provided as a Source Data file.

We anticipated that the antibodies induced by the VLPs would be cross-reactive with VACV proteins, as there are only 5, 6, and 8 amino acid differences for MPXV M1, A35, and B6 compared to their VACV homologs L1, A33, and B5, respectively. This cross-activity allowed us to take advantage of mouse models established for VACV^50^, enabling experiments to be performed with common inbred mice at a lower biosafety level than with MPXV. BALB/c mice were primed and then boosted twice at 3-week intervals with individual monovalent VLPs, combinations of the monovalent VLPs, the chimeric multivalent VLP containing all three proteins, a combination of all three SPs, or unconjugated SC-mi3 as a negative control. There were 2- to 3-times more antigens in the SPs than in the VLPs as the antigens of the latter were fused to SC-mi3, and the same weights of SPs and VLPs were used for immunizations. In each case, the samples were mixed with an equal volume of AddaVax adjuvant prior to injection. As a positive control, mice were immunized with MVA. Serum was obtained for analysis at 3 weeks after each immunization. Similar titers of binding antibodies to VACV L1 were detected in pooled serum obtained from mice at 3 weeks after priming with the M1 VLP alone or M1 VLPs in combination with other VLPs, and the titers increased after each boost (Fig. 2a). Antibodies to L1 were below detection after the prime with SPs but increased after the boosts, though not to the levels achieved with VLPs, even though the SPs contained more antigen. The level of antibody to L1 induced by the VLPs was one to two logs higher than that produced by MVA (Fig. 2a). Similar patterns with the highest values achieved with VLPs were obtained when analyzing binding antibodies to VACV A33 (Fig. 2b) and VACV B5 (Fig. 2c). The chimeric VLP, containing all three proteins, elicited binding antibodies similar to the combination of monovalent VLPs. Although the binding antibody titers to L1, A33, and B5 induced by MVA were more than a log lower than the titers in sera from mice immunized with VLPs, it should be mentioned that MVA induces antibodies to many additional proteins^51^.

Next, we determined anti-VACV neutralization titers of serum from individual mice. Of the three protein components, only anti-M1 neutralizes MVs, as A35 and B6 are components of the EV membrane. The 50% neutralization titers (NT50s) were determined by flow cytometry using a previously described assay^52^ in which neutralizing antibody inhibits infection of cells by a recombinant VACV expressing green fluorescent protein (GFP). After the prime immunization the difference in neutralization between the sera from mice that received the combination of VLPs and the sera from mice that received the combination of SPs or MVA was highly significant, whereas there were smaller differences from those that received either individual VLPs or the chimeric VLP (Fig. 2d). The serum titers increased after the first boost but mostly plateaued after the second. The NT50 values determined for sera of mice immunized with the combination of VLPs remained higher than the SPs after each boost, but the difference diminished. In contrast, the difference between the VLPs and MVA was highly significant even after the second boost.

As antibodies to A35 and B6 do not neutralize MVs, a virus spread comet assay was used to determine the inhibitory activity of serum following immunization with those EV protein VLPs. In this assay, immune serum is added to the liquid overlay after cells are infected with the IHD-J strain of VACV, which produces large numbers of EVs. Two days later, the cell monolayers are stained with crystal violet, and satellite plaques appear as non-staining comets due to the cytopathic effects of VACV. Pooled serum from mice immunized with B6 or A35 + B6 greatly reduced the size of comets compared to the control serum obtained from mice that received the unconjugated SC-mi3, whereas the anti-A35 serum exhibited less inhibition (Fig. 2e).

To evaluate cross-protection by the MPXV VLPs and homologous protection by MVA, immunized BALB/c mice were challenged with a 10^6^ plaque-forming units (PFU) (~10× the 50% lethal dose) of VACV strain Western Reserve (WR), and weight loss and survival were determined. According to our animal protocol, a reduction to 70% of the starting weight automatically triggers euthanasia. Control mice that received only the unconjugated SC-mi3 rapidly lost weight and succumbed, whereas all immunized mice survived although with varying weight loss (Fig. 2f). Mice immunized only once with the combination of three VLPs lost more weight than those boosted once or twice (*p* < 0.0001) calculated for combined days 5, 6 and 7 by one-way ANOVA with multiple comparisons, whereas there was no significant difference between one and two boosts. Also, mice boosted with the three SPs lost slightly more weight from days 5–8 than those boosted with the three VLPs but the difference did not reach statistical significance. Although the neutralization titers induced by MVA were consistently lower than those induced by the VLPs, the mice were still protected against weight loss (Fig. 2f).

To assess the contributions of the individual antigens, the outcomes for mice that received three immunizations with single monovalent VLPs or pairs of monovalent VLPs were determined as part of the above experiment but plotted separately for clarity (Fig. 2g). All mice survived except for one mouse that received only the A35 VLP. However, mice immunized with the individual M1, A35 or B6 VLPs or with A35 plus B6 VLPs lost appreciable weight, whereas mice that received M1 plus A35 or M1 plus B6 VLPs lost significantly less weight, (*p* < 0.0001) calculated for the sum of days 5, 6, and 7 calculated by one-way ANOVA with multiple comparisons, than each of the other groups but were not significantly different from each other. The finding that the most protective vaccines contained at least one MV VLP and one EV VLP was consistent with prior studies with SPs^48^ and could provide the minimal components of a recombinant vaccine.

### Immunogenicity of MPXV VLPs and protection in a lethal MPXV challenge model

**Fig. 3:**
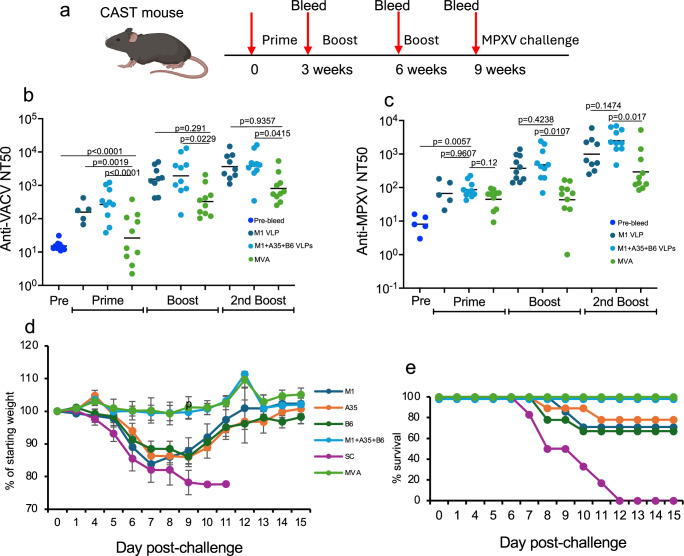
Vaccine-induced antibodies and protection of CAST mice against a lethal MPXV challenge. **a** CAST mice (*n* = 10) per group, combined from two experiments, were immunized IM three times at 3-week intervals and bled prior to each immunization. Mice were challenged IN with 10^4^ PFU of clade IIa MPXV at 3 weeks after the third immunization. **b** Anti-VACV and **c** anti-MPXV neutralization titers were determined in duplicate in individual mice prior to immunization (Pre-bleed) or at 3 weeks after receiving 1 (Prime), 2 (Boost), or 3 (2nd Boost) IM immunizations with 2 µg of M1 VLP; a combination of 2 µg each of M1 + A35 + B6 VLPs; or 10^7^ PFU of MVA. Bars are geometric means. Significance was determined with one-way ANOVA and a multiple comparison test. **d** Weight loss and **e** survival of individual CAST mice challenged IN with 10^6^ PFU of MPXV clade IIa at 3 weeks after third immunization with indicated VLPs, SPs, MVA, or control unconjugated SC-mi3. Due to accidental loss, there were 9 immunized mice in the A35 VLP, B6 VLP, and MVA; 8 in the M1 + A35 + B6 VLPs; 7 in the M1 VLPs; and 6 in the SC-mi3 groups. Bars are SD. Source data are provided as a Source Data file. Created in BioRender. Moss (2025) https://BioRender.com/xkyulng.

The Castaneous/EiJ (CAST) wild-derived inbred mouse strain is susceptible to lethal infection with MPXV subclades Ia and IIa, whereas common genetically related inbred mouse strains are more resistant^53,54^. CAST mice were primed and boosted with M1 VLP, M1 + A35 + B6 VLPs, or MVA at 3-week intervals, and serum was obtained prior to each immunization (Fig. 3a). The anti-VACV (Fig. 3b) and anti-MPXV clade Ia (Fig. 3c) neutralization titers of mice immunized with the combination of VLPs were similar to those attained with M1 VLP alone and significantly higher than those attained with MVA.

Next, we compared the protection afforded by the MPXV clade Ia protein VLPs and MVA to challenge with a subclade IIa MPXV. Following the challenge, the control CAST mice that received unconjugated SC-mi3 rapidly lost weight and succumbed to the infection, whereas mice that were immunized with the combination of all three VLPs or MVA lost no weight and survived (Fig. 3d, e). However, mice immunized with individual VLPs lost considerable weight, and some of these mice succumbed to the infection. Thus, the combination of M1, A35, and B6 VLPs, like MVA immunization, is protective against both VACV and MPXV subclade IIa in a lethal mouse model.

### VLP and MVA vaccines reduce virus replication at the IN site of inoculation and prevent spread

**Fig. 4:**
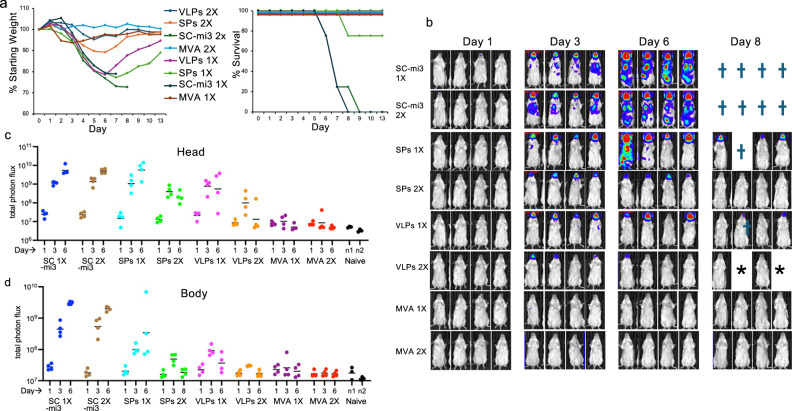
Reduction of VACV replication at the IN site of inoculation and spread. **a** BALB/c mice (*n* = 4 per group) received 1 (1×) or 2 (2×) IM immunizations with a mixture of M1, A35, and B6 VLPs or SPs, MVA, or SC-mi3 control and challenged 3 weeks later IN with 10^6^ PFU of WRvFire. Weight loss and survival were determined. Bars represent SD. **b** Mice in (**a**) were imaged following IP injection of luciferin on indicated days. BLI is depicted by a pseudocolor scale with intensity increasing from blue to red. **c**, **d** Photon flux measurements of mice from (**a**). Regions of interest were drawn to distinguish luminescence from the head (**c**) and from the body (chest and abdomen) (**d**). Total photon flux is plotted as photon/s/cm^2^/sr. Symbols: †, death due to infection; *, anesthesia death. Significance between control SC-mi3 (combined 1× and 2×) and vaccine-immunized mice determined for day 6 by the Kruskal–Wallis/Dunn’s multiple comparison test. Source data are provided as a Source Data file.

The ability of the vaccines to reduce infection at the site of inoculation and prevent spread to the chest and abdomen was determined using WRvFire, a recombinant VACV that retains virulence and expresses firefly luciferase, enabling non-invasive live imaging of individual animals on successive days^55,56^. BALB/c mice were immunized twice at three-week intervals with control SC-mi3, the mixture of three VLPs, the mixture of three SPs, or MVA, and one group was challenged three weeks after the first immunization with WRvFire, and another group 3 weeks after the second immunization. The challenge results with WRvFire were similar to those already shown for WR. Control mice that received one or two injections of unconjugated SC-mi3 rapidly lost weight and did not survive following IN inoculation with VACV WRvFire (Fig. 4a). All mice receiving one or two immunizations with the combination of three SPs lost weight, though all but one mouse recovered. Mice receiving one immunization with the combination of three VLPs also lost weight but survived and those receiving a VLP or MVA boost lost little or no weight (Fig. 4a). Statistical analysis calculated by one-way ANOVA with multiple comparisons for weights on days 5–7 indicated significantly better protection after the boosts compared to a single vaccinations for both SPs and VLPs (*p* < 0.0001). Although mice immunized with VLPs lost less weight and recovered more rapidly than mice immunized with SPs, the differences were not significant for the sum of days 5–7.

The mice of Fig. 4a were injected with luciferin on successive days after challenge, and bioluminescence (BLI) was visualized. The same camera settings, chosen to prevent image saturation, were used for all mice. As luciferase has a half-life of about 2 h in animal cells^57^, BLI correlates with active virus replication. Luker and Luker^58^ described a significant correlation between BLI and plaque-forming units using a recombinant VACV. A caveat, is that detection is more efficient from superficial tissues than deep issues because of light quenching^58^. For this reason, comparisons are made between the same regions of different mice rather than between different areas of a mouse. Mice that had received unconjugated SC-mi3 exhibited BLI in the head and chest on day 3 that spread to the abdomen by day 6, followed by death (Fig. 4b). Mice immunized once with the SPs also exhibited intense BLI in the head and some in the body that was cleared for all but one by day 8. However, a second immunization with SPs reduced BLI in the head and prevented spread to the body. One immunization with the VLPs prevented spread to the body, and a second immunization also greatly reduced BLI in the head. BLI in the head and bodies was below detection in mice that received MVA.

The BLI data were quantified by measuring photons of light emitted per unit of time from a defined area (photon flux). The photon flux was determined separately for the head and body regions of individual mice. In the control mice receiving unconjugated SC-mi3, photon flux of the heads including the site of VACV inoculation, were detected on day 1 and increased on days 3 and 6 (Fig. 4c). Compared to the control mice on day 6, the photon flux decreased after the second immunization with SPs but did not reach the 95% significance level (Fig. 4c). However, the photon flux was reduced after the first immunization with VLPs and reached significance compared to the controls after the second immunization. After one or two immunizations with MVA, the photon flux was significantly reduced (Fig. 4c).

Photon flux in the bodies paralleled that in the heads of control and immunized mice (Fig. 4d). In the control mice receiving unconjugated SC-mi3, photon flux in the body was slightly above baseline on day 1 and increased greatly on days 3 and 6 prior to death. Compared to the control mice on day 6, photon flux was decreased after the first immunization with SPs and VLPs and reached 95% significance after the second immunization, and was significantly reduced after one or two immunizations with MVA. Overall, VACV was cleared most rapidly in mice immunized with MVA, followed by VLPs and SPs.

### VLP vaccine inhibits virus replication at the IR site of inoculation

**Fig. 5:**
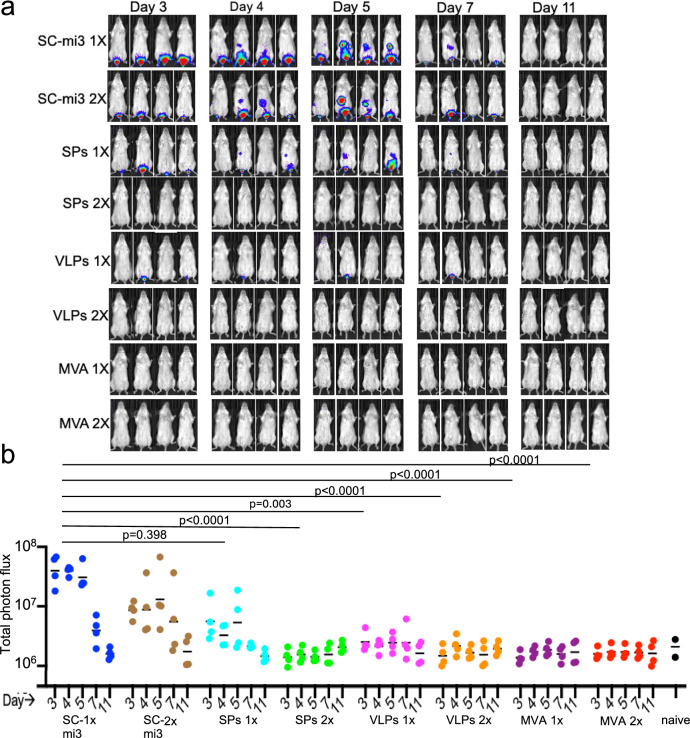
Reduction of VACV replication at the IR site of inoculation and spread. **a** BALB/c mice (*n* = 4 per group) received two IM immunizations with SC-mi3 control, mixture of three SPs, mixture of three VLPs, or MVA, and were challenged 3 weeks later with 10^6^ PFU of WRvFire IR. Imaging of individual mice was performed on days between 3 and 11, with intensity of BLI increasing from blue to red. **b** A region of interest was drawn around the rectum to determine luminescence from that area. Total photon flux is plotted as photon/s/cm^2^/sr. Significance determined for comparison of the combined control SC-mi3 1× and 2× on days 3–5 vs each immunized group on combined days 3–5 by Kruskal–Wallis/Dunn’s multiple comparison test. Source data is provided as a Source Data file.

Transmission of MPXV during the 2022 global outbreak involved sexual activity between men with IR infections and mucosal spread, and there is also evidence for increased sexual transmission of the more pathogenic clade I MPXV strains^12,59^. For these reasons, we determined the ability of the VLP vaccine administered IM to inhibit virus replication at the IR site of inoculation. Immunized mice were inoculated IR with WRvFire and imaged from days 3–11. The controls that received unconjugated SC-mi3 exhibited intense BLI in the rectal area from days 3–5 with some abdominal spread that was resolved between days 7 and 11 (Fig. 5a). Reduced BLI was detected in mice that received one immunization with the combination of SPs and no BLI was detected in mice that received two. Only one of five mice immunized once with the combination of VLPs had detectable BLI, and none was detected in mice that received two immunizations with the VLPs or one or two with MVA. Photon flux measurements confirmed the impressions of the images (Fig. 5b). Significance was determined by comparing the combined photon flux data of the two control SC-mi3-immunized groups for days 3–5 with the combined 3–5 days data of each of the immunized groups. All of the groups, except for mice receiving a single immunization with SPs, had significantly lower photon flux than the controls. These data demonstrated that the vaccines administered IM could inhibit replication and spread from IR, as well as IN inoculation sites, and that VLPs are superior to SPs.

### Neutralizing and VACV protective antibodies induced by immunization of rhesus macaques

**Fig. 6:**
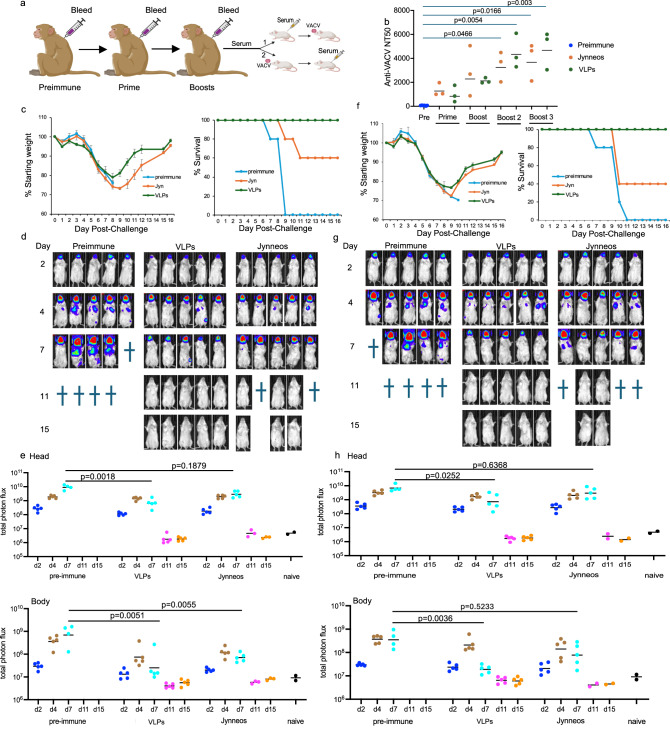
Induction of anti-VACV neutralizing and protective antibodies in rhesus macaques. **a** Rhesus macaque (*n* = 3 per group) received a total of 30 µg of a mixture of the three monovalent VLPs or 0.5 ml of Jynneos vaccine subcutaneously at 3-week intervals. BALB/c mice were injected IP with pooled serum obtained after the third immunization either (1) one day before or (2) one day after IN challenge with WRvFire. **b** Anti-VACV neutralizing titers were determined in duplicate for serum of individual macaques obtained prior to immunizations (Preimmune) or 3 weeks after each immunization. Bars indicate geometric means. Significance between preimmune and immune sera was determined by Kruskal–Wallis/Dunn’s multiple comparison test. **c** BALB/c mice (*n* = 5 per group) were injected IP with 0.5 ml of pooled preimmune serum or serum pooled from animals following boost 3 with Jynneos or VLPs. Weight loss and survival were determined following the IN challenge of BALB/c mice with 10^5^ PFU of WRvFire as in scheme (1) of (**a**). Bars represent SD. **d** BLI of BALB/c mice that were passively immunized one day before challenge, as in (**c**). †, death. **e** Total photon flux from same mice imaged in (**d**). Regions of interest were drawn to distinguish luminescence emanating from the head or from the body (chest and abdomen). Total photon flux is plotted as photon/s/cm^2^/sr. Significance between the preimmune and immune values was determined on day 7 by Kruskal–Wallis/Dunn’s multiple comparison test. **f** Like (**c**) except that serum was injected one day after mice were challenged with WRvFire as depicted in scheme (2) of (**a**). **g** Like (**d**) except that mice from (**f**) that received serum one day after infection were imaged. **h** Like (**e**) except that photon flux was determined in mice from (**g**). Source data is provided as a Source Data file. Created in BioRender (2025) https://BioRender.com/sv7gwy2.

The next series of experiments had several objectives. First, there was a comparison of neutralizing antibody titers induced in NHPs by the VLPs and the Jynneos vaccine. Second, it was determined whether serum antibodies alone could provide protection against VACV and MPXV challenge. Third, was whether the serum antibodies need to be administered before challenge or whether they could be added afterwards to reduce infection (Fig. 6a).

For vaccination of rhesus macaques, we used the human dose of Jynneos (formulated to contain 0.5 × 10^8^ to 3.95 × 10^8^ infectious units) in 0.5 ml administered subcutaneously (~10× the mouse dose of MVA). For the three VLPs, we combined 10 µg of each so that the combination was 30 µg (5× the mouse dose) mixed with AddaVax and administered subcutaneously instead of IM. The anti-VACV neutralization titers in serum from the three animals in each group were above the pre-immunization values after the prime and increased substantially with each boost (Fig. 6b). After the second and third boosts, the mean NT50s for the sera of the monkeys vaccinated with Jynneos or VLPs were significantly higher than the pre-immune sera, and the titer of the VLP serum was about twice that of the Jynneos serum.

In part (1) of this experiment (Fig. 6a), 0.5 ml of pooled serum from macaques immunized with Jynneos or VLPs was injected intraperitoneally (IP) into BALB/c mice one day before IN infection with WRvFire. The anti-VACV NT50 titers determined by bleeding mice one day after receiving serum were ~200 and ~400 for the mice receiving Jynneos and VLP serum, respectively, consistent with the difference shown in Fig. 6b. Following IN challenge with 10^5^ PFU of WRvFire, the control mice that received preimmune serum all lost weight and succumbed to the infection (Fig. 6c). The mice that received anti-VLP serum lost weight but all recovered, whereas 2 of 5 mice challenged after receiving anti-Jynneos serum lost more weight and succumbed. Live animal imaging of BALB/c mice that received pre-immune serum and were challenged with WRvFire exhibited BLI in the heads on day 2 that spread to the bodies by day 4 and increased in intensity by day 7 before succumbing to the infection (Fig. 6d). Mice that received anti-VLP serum had lower BLI in the head and minimal in the bodies that was cleared by day 11. The BLI of mice receiving anti-Jynneos serum was intermediate between those that received pre-immune and anti-VLP serum, but those that survived also cleared the infection. These results were confirmed by photon flux measurements: in the heads on day 7, the difference between controls and the mice was significant for those receiving anti-VLP serum but not anti-Jynneos serum; in the bodies on day 7, the significance was greater between the controls and the mice receiving anti-VLP serum than anti-Jynneos serum (Fig. 6e).

In part (2) of this experiment, the mice were first infected, and the serum was injected one day later after establishment of the infection. Again, mice that received control preimmune serum died, while mice that received anti-VLP serum lost weight but all recovered (Fig. 6f). In contrast, 3 of 5 mice that had received anti-Jynneos serum died. The imaging data obtained with mice that received serum one day after challenge was qualitatively similar to that obtained for mice that received serum one day before (Fig. 6g). However, the photon flux in the heads and bodies of mice receiving preimmune serum and anti-VLP serum was statistically significant, whereas the difference between mice receiving preimmune serum and anti-Jynneos serum was not (Fig. 6h). Thus, the anti-VLP serum was protective when administered 1 day before or 1 day after a VACV challenge, whereas the anti-Jynneos serum was less protective consistent with the lower neutralization titer.

### Neutralizing and MPXV protective antibodies induced by immunization of rhesus macaques

**Fig. 7:**
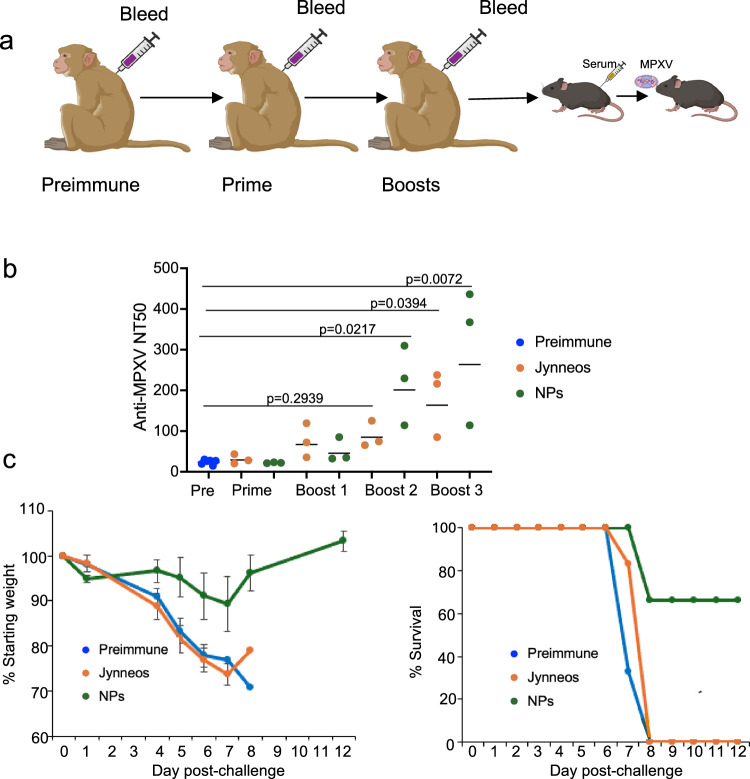
Induction of anti-MPXV neutralizing and protective antibodies in rhesus macaques. **a** The pooled serum from rhesus macaques immunized in Fig. 6 with a total of 30 µg of a mixture of the three monovalent VLPs or 0.5 ml of Jynneos vaccine subcutaneously at 3-week intervals was injected into CAST mice one day before challenge with 10^4^ PFU of MPXV clade IIa. **b** Anti-MPXV neutralizing titers were determined in duplicate for serum of individual macaques obtained prior to immunizations (Preimmune) or 3 weeks after each immunization with Jynneos or VLPs. Bars indicate geometric means. Significance between preimmune and immune sera determined by Kruskal-Wallis/Dunn’s multiple comparison test. **c** CAST mice (*n* = 6 per group) were injected IP with 0.5 ml of pooled preimmune serum or serum pooled from animals following boost 3 with Jynneos or VLPs. Weight loss and survival were determined following the IN challenge of CAST mice with 10^5^ PFU of MPXV. Bars represent SD. Source data is provided as a Source Data file. Created in BioRender. Moss (2025) https://BioRender.com/u3e5g0y.

The anti-Jynneos and anti-VLP macaque serum used in the previous experiment was also tested for neutralization of the homologous MPXV clade Ia (Fig. 7a). The MPXV serum titers induced by VLPs were significantly higher than the preimmune titers after the second and third boosts, whereas the titers induced by Jynneos only reached significance after the third boost and were lower than those of anti-VLP serum.

To evaluate protection against MPXV, CAST mice received 0.5 ml of pre-immune or anti-Jynneos or anti-VLP serum one day prior to challenge with 10^5^ PFU of MPXV clade IIa. The control mice, as well as the mice injected with anti-Jynneos serum, rapidly lost weight and died, whereas 4 of the 6 mice injected with anti-VLP serum recovered, consistent with the higher neutralizing antibody titer of the former (Fig. 7c). Thus, the serum from macaques immunized with VLPs constructed with clade Ia MPXV proteins provided cross-protection to challenge with VACV or clade IIa MPXV.

## Discussion

The Jynneos vaccine used during the recent mpox outbreak is comprised of the highly attenuated non-replicating MVA strain of VACV, and is used at a high dose that is difficult to manufacture in bulk^60,61^. Early attempts to produce a safe and effective inactivated VACV vaccine were unsuccessful: despite the production of high levels of neutralizing antibodies, mice developed severe disease upon VACV challenge^62^ even when attempts were made to include EVs^63^. Here we describe the construction of VLPs that are decorated with both MV and EV proteins and provide good protection in animal models.

We used a SpyCatcher scaffold with 60-antigen coupling sites for display of the MPXV clade Ia M1 MV protein and A35 and B6 EV proteins in individual or chimeric VLPs. At equivalent protein concentrations with AddaVax adjuvant, the chimeric VLP induced similar levels of binding and neutralizing antibodies as the combination of individual VLPs. In contrast to the VLPs, antibodies were undetectable after the prime with SPs and did not reach equivalent levels after boosting, even though the SPs contained 2- to 3-times more antigen than the VLPs. The more rapid immune response achieved with the VLPs compared to SPs would be particularly important for containing outbreaks. Additionally, the anti-B6 and anti-A35 antibodies induced by the VLPs reduced the spread of extracellular virus in an in vitro assay.

Protection was first tested in a well-characterized mouse IN infection model^50^ using a challenge dose of VACV strain WR that was 10× the 50% lethal dose. A single immunization with the VLP vaccine completely protected against death, although transient weight loss occurred; weight loss was minimal after one or two boost vaccinations. By immunizing mice with VLPs containing single proteins, we confirmed that M1, A35, and B6 each contributed to protection, justifying their presence in the combined vaccine. CAST mice were used to determine protection against MPXV, as the classic inbred mouse strains are more resistant^53^. CAST mice are immunologically competent and make strong antibody and T-cell responses but have low NK cell activity, which contributes to their susceptibility to MPXV and other orthopoxviruses^64^. As with BALB/c mice, the highest neutralizing antibody titers were achieved with the VLPs, and the combination of the three protected against weight loss and death following IN infection with a subclade IIa MPXV. Thus, subclade Ia MPXV protein VLPs induced antibodies that neutralized both MPXV subclade Ia and VACV and cross-protected mice against both VACV and subclade IIa MPXV challenges.

To evaluate effects on virus replication, we used WRvFire, a recombinant VACV that expresses firefly luciferase, allowing live animal imaging of infection. The light emitted is directly proportional to the number of enzyme molecules and because luciferase has a short half-life, BLI is a measure of active virus infection^58^. In unvaccinated mice inoculated IN with WRvFire, BLI in the head and chest was detected by day 3 and spread to abdominal organs by day 6. In contrast, mice vaccinated with VLPs had transient BLI in the head that did not spread to the chest or abdomen, whereas there was greater BLI in mice vaccinated with SPs. Although we did not measure the effects of vaccination on transmission, it is likely that reduced levels of virus at the site of inoculation would mitigate transmission. As human-to-human MPXV transmission can occur by close contact and was mainly spread by men who have sex with men during the recent global outbreak^65^, we demonstrated that the VLP vaccine also prevented an IR infection with WRvFire. Thus, IM administration of VLPs protects against multiple routes of infection.

Lastly, we compared the immunogenicity of a 30 µg dose of the VLP vaccine with the human dose of Jynneos in a macaque model. As with mice, the VLPs induced higher anti-VACV and anti-MPXV neutralizing antibodies than MVA. As a facility suitable for challenging the macaques with MPXV was unavailable, we tested the antisera by passive transfer in mice. Prior studies demonstrated the functionality of primate IgG in mice^66^ and passive transfer of monkey or human immune serum was shown to protect mice against bacterial and viral infections^67–69^. In our study, all mice challenged with VACV one day after or one day before receiving macaque anti-VLP serum survived, whereas anti-Jynneos serum, which had a lower neutralizing titer, provided only partial protection. Moreover, only the mice that received macaque anti-VLP serum survived an MPXV challenge. However, following active immunization of mice, the MVA and VLP vaccines were similarly protective despite the lower neutralizing activity of the anti-MVA serum, suggesting that non-neutralizing antibodies or T cells might contribute to protection by MVA in mice.

In conclusion, we showed that a VLP vaccine containing three MPXV clade Ia antigens induces higher neutralizing antibodies and better cross-protection against a virulent strain of VACV and a subclade IIa MPXV than the same SP antigens in mouse models. In addition, the VLP vaccine induced higher neutralizing and protective antibodies than the current Jynneos vaccine in NHPs. Using similar MPXV antigens, mRNA vaccines^24^ and the current VLP vaccine induced comparable levels of neutralizing antibodies and provided protection in animal models. Further studies are needed to determine whether the repetitive array of antigens on VLPs confers additional benefits.

## Methods

### Study design

The study was undertaken to evaluate the immunogenicity and protective efficacy of VLP vaccine candidates containing 1–3 highly conserved MPXV subclade Ia proteins (M1, A35, B6) in murine challenge models. Binding antibodies to the VACV homologs of the individual protein components and neutralizing activities to VACV strain WR and MPXV subclade Ia were determined. The immunized mice were challenged with VACV strain WR or MPXV subclade IIa by respiratory or mucosal routes. The primary endpoints of weight loss and survival were determined by blinded investigators. Luciferase-expressing VACV was used to monitor infection at the site of inoculation and virus spread to the body. Contributions of the individual protein components of the vaccine to protection were determined, and comparisons were made to both SPs and to the MVA vaccine. The neutralizing antibody responses of NHPs to the VLPs and the licensed Jynneos vaccine were compared, and their protective efficacy was determined by passive transfer of serum to mice that were challenged with VACV strain WR or MPXV clade IIa. Animal sample sizes and VACV and MPXV challenge doses were determined by pilot experiments. Animals were randomly assigned to each group. Each experiment included both negative controls and MVA as a positive control. Sizes of groups and technical/biological replicates are indicated in the figure legends.

### Biosafety

Procedures with infectious VACV were carried out at Biosafety Level 2 and with MPXV clades Ia and IIa in a select agent Biosafety Level 3 laboratory by trained and vaccinated investigators using protocols approved by the NIH Institutional Biosafety Committee. Sample inactivation and removal were performed according to standard operating protocols approved by the local Institutional Biosafety Committee.

### Mice

Female 5- to 6-weeks old BALB/cAnNTac and CAST/EiJ mice were purchased from Taconic Biosciences (Germantown, NY) and Jackson Laboratories (Bar Harbor, ME), respectively, and 3–5 mice were maintained in small, ventilated microisolator cages. Male mice were not used as they would need to be housed in separate cages, which were not available, to prevent aggressive behavior. Experiments and procedures (animal study protocol LVD-29E) were approved by the National Institute of Allergy and Infectious Diseases (NIAID) Animal Care and Use Committee according to standards set forth in the NIH guidelines, Animal Welfare Act, and US Federal Law. Euthanasia was carried out when weight loss reached 30% using carbon dioxide inhalation followed by cervical dislocation in accordance with the American Veterinary Medical Association (AVMA) guidelines (2013 Report of the AVMA panel on euthanasia).

### NHPs

Rhesus macaques were housed and cared for at the NIH Animal Center, under the supervision of the Association for the Assessment and Accreditation of Laboratory Animal Care (AAALAC)-accredited Division of Veterinary Resources, and as recommended by the Office of Animal Care and Use Nonhuman Primate Management Plan. Husbandry and care met the standards set forth by the Animal Welfare Act, Animal Welfare Regulations, as well as The Guide for the Care and Use of Laboratory Animals (8th Edition), as detailed previously in ref. ^70^. The NIAID Division of Intramural Research Animal Care and Use Program, as part of the NIH Intramural Research Program, approved all experimental procedures (animal study protocol LVD 26).

### Viruses

VACV WR (ATCC), VACV WRvFire^71^, clade IIa MPXV-USA-2003-044^53^, VACV WR-GFP^52^, clade Ia MPXV Z-1979-GFP^24^, MVA^72^ (seed virus for ACAM3000 GenBank: AY603355.1), VACV-IHDJ^73^ were previously described and grown in BS-C-1 cells (CCL-26, ATCC).

### Expression and purification of proteins

pET28a-SpyCatcher003-mi3 (GenBank MT945417, AddGene 159995) and its expression and purification from *Escherichia coli* were previously described in ref. ^49^. M1, A35, and B6 protein sequences were derived from MPXV-ZAI-1979-005 and chemically synthesized by Twist Biosciences (South San Francisco, CA) with mammalian codon optimization, *N*-glycosylation site mutations, deletion of the TM domain, addition of the influenza H7 strain hemagglutinin signal peptide, and additions of 6-histidines and a SpyTag. The DNA was inserted between the XbaI-NheI restriction enzyme sites of pcDNA3.1. Plasmids were transfected with ExpiFectamine293 into Expi293F cells (ThermoFisher, Waltham, MA) following the manufacturer’s protocol. The medium was collected 5 days after transfection and centrifuged at 4 °C for 15 min at 3000×*g* to pellet cells and debris. Typical protein yields were 5–7 mg per 25 ml. Clarified supernatants were adjusted to contain 5 mM imidazole and incubated with Ni-NTA affinity agarose (Qiagen, Germantown, MD) for 2 h at 4 °C with gentle agitation. The mixture was poured into an Econo-Column (BioRad, Hercules, CA), and 2 × 12 column bed volumes of wash buffer (20 mM imidazole, 300 mM NaCl, and 20 mM Tris/HCl, pH 7.5) were applied, followed by 12 column-bed volumes of 200 mM and 500 mM imidazole. Fractions were analyzed by sodium dodecyl sulfate polyacrylamide gel electrophoresis, stained with Coomassie blue, blotted, and probed with mouse anti-histidine antibody (clone AD 1.1.10, BIO-RAD) and visualized with HRP-conjugated goat anti-mouse IgG (Catalog # 31430, ThermoFisher). Fractions containing the proteins of interest were pooled, concentrated, and buffer exchanged into phosphate-buffered saline (PBS) (Quality Biological, Gaithersburg, MD) using Vivaspin-20 30 kDa tubes (Cytiva, Marlborough, MA) and then passed through a 0.22 µm filter. Protein concentrations were determined using a Qubit broad-range protein assay kit (ThermoFisher).

### Conjugation of proteins and purification of VLPs

SpyTagged proteins were conjugated at 4 °C for 16 h at a 5-fold molar excess to 2 µM SpyCatcher003-mi3 in PBS (pH 8.0). Aggregates were removed by centrifugation at 16,900×*g* for 30 min at 4 °C, and the supernatant was concentrated with Vivaspin-20 100 kDa tubes to 0.5 ml and then centrifuged at 3000×*g* for 30 min to remove aggregates. Supernatants containing particles were purified by size exclusion chromatography on a 10/300 GL column of Superdex 200 equilibrated with PBS using an ÄKTA Pure 25 (GE Life Sciences, Marlborough, MA) with a 0.25 ml/min flow rate.

### Immunization of mice

Mice received 2 µg of an individual VLP or SP, mixtures containing 2 µg of each VLP or SP, 6 µg of the chimeric VLP, or 2 µg or 6 µg of unconjugated SC-mi3 in PBS emulsified with an equal volume of AddaVax adjuvant (InvivoGen, San Diego, CA). In each case, the amount of protein injected is the quantity determined using the Qubit assay kit, without taking into account the proportions of antigen and SC-mi3. The proteins were injected IM in a total volume of 50 µl. MVA (10^7^ PFU) in 50 µl of PBS with 0.5% (*w*/*v*) bovine serum albumin was administered IM. Mice were boosted at 3-week intervals and bled prior to each immunization to obtain serum.

### Binding and neutralization assays

Purified baculovirus-produced VACV soluble LI, A33, and B5 proteins^48^, a kind gift of Gary Cohen (University of Pennsylvania), were used for ELISA. 50 ng of protein was adsorbed overnight to each well of a 96-well high-binding plate (ThermoFisher). The wells were washed with 1× buffer (prepared from 10× buffer: 270 g NaCl and 30 ml Tween-20 in 3 l H_2_O), blocked for 2 h at room room temperature with 100 µl of 0.2 ml Tween-20 and 5 g nonfat dry milk in 100 ml PBS, washed again, and incubated with 2-fold dilutions of serum for 1 h. After washing, plates were incubated with horseradish peroxidase-conjugated goat anti-mouse IgG at a 1:2000 dilution (Thermo Fisher). Blue BM substrate (Millipore-Sigma, St. Louis, MO) was then added to each well. Spectrophotometric measurements were made at A370 and A492 using a Synergy H1 plate reader with Gen5 analysis software (Agilent Technologies, Santa Clara, CA). Final end point titers (1/*n*) for each sample were determined as four-fold above the average optical density of those wells not containing primary antibody. Neutralization assays were carried out as described previously using VACV WR-GFP and MPXV Z-1979-GFP in a 96-well plate flow cytometric assay^24,52^. NT50 values were calculated by non-linear regression with GraphPad Prism (Reston, VA). The limit of detection (LOD) was determined by taking 1.96 standard deviation of the mean titer of the control samples.

### Comet spread assay

Antibody inhibition of EV spread was determined essentially as described in ref. ^74^. BS-C-1 monolayers in 12-well plates were incubated with 30 PFU of VACV-IHDJ for 1 h at 37 °C in Eagle’s minimum essential medium (MEM) (Quality Biological) supplemented with 2% fetal bovine serum. After 1 h, the medium was aspirated, and the monolayers were washed with fresh medium to remove free virus. Cell monolayers were then overlaid with 1:50 diluted, heat-inactivated pooled immune mouse serum, incubated at 37 °C for 48 h at a slight tilt, and then fixed and stained with crystal violet.

### VACV and MPXV infection of immunized mice

VACV WR and VACV WRvFire^71^ were purified by sucrose gradient sedimentation, and 10^5^ or 10^6^ PFU, as indicated in the figure legends, were inoculated IN into 20 µl of PBS with 0.05% (*w*/*v*) bovine serum into one nostril. For IR infection, 10^6^ PFU of WRvFire was administered IR in a volume of 15 µl by inserting a pipette tip attached to a syringe into the rectum. The animals were held upside down for 2 min to prevent leakage of the material. Purified MPXV USA-2003 was administered IN at a dose of 10^4^ PFU as described for WRvFire.

### BLI and photon flux

Mice lightly anesthetized with isoflurane were injected IP with Xenolight D-luciferin substrate (150 μg/g body weight) before imaging with an IVIS Lumina LT series III system (Perkin Elmer, Waltham, MA) as described in ref. ^55^. Living Image Software (Perkin Elmer) was used for image acquisition and analysis. Photon flux was measured by outlining the head and body separately as regions of interest and quantifying light emission in photons per second per square centimeter per steradian.

### Immunization of rhesus macaques

Two male and one female rhesus macaques weighing 11.4 kg, 13.7 kg, and 9.4 kg, respectively, were immunized subcutaneously with 0.5 ml of vaccine comprised of 10 µg of each of the three VLPs in PBS emulsified with an equal volume of AddaVax adjuvant and boosted at 3-week intervals. Three males weighing 10.5 kg, 12.8 kg, and 12.8 kg were immunized subcutaneously with 0.5 ml of Jynneos vaccine and boosted at 3-week intervals.

### Passive serum transfer

Heat-inactivated macaque serum obtained prior to immunization or after priming and boosting three times with VLPs or with Jynneos were separately pooled and 0.5 ml was injected IP into BALB/c mice, which were bled one day later to determine neutralizing antibodies, and challenged with 10^5^ PFU of WRvFire either 1 day before or 1 day after serum transfer. CAST mice also received 0.5 ml of pooled serum and were challenged with 10^5^ PFU of clade IIa MPXV-USA-2003-04. Mice were observed, weighed, and imaged over a 2-week period.

### Data analysis

Significance of survival and photon flux was determined by one-way ANOVA and Kruskal–Wallis/Dunn’s multiple comparison tests using GraphPad Prism v10.

### Reporting summary

Further information on research design is available in the Nature Portfolio Reporting Summary linked to this article.

## Supplementary information

Peer Review file

Reporting Summary

## Source data

Source Data

## Data Availability

Source data are provided with the paper, and additional information and unique materials are available upon request. MPXV Z-1979-GFP was deposited in BEI Resources and is available upon request. Source data are provided with this paper.

## References

[1] Moss, B. Understanding the biology of monkeypox virus to prevent future outbreaks. *Nat. Microbiol.* **9**, 1408–1416 (2024). 38724757 10.1038/s41564-024-01690-1

[2] Satheshkumar, P. S. & Damon, I. in *Fields Virology: DNA Viruses*, Vol. 4 (eds Howley, P. M., Knipe, D. M., Cohen, J. L., & Damania, B. A.) Ch. 17, 614–640 (Wolters Kluwer, 2021).

[3] Bunge, E. M. et al. The changing epidemiology of human monkeypox—a potential threat? A systematic review. *PLoS Negl. Trop. Dis.* **16**, e0010141 (2022). 35148313 10.1371/journal.pntd.0010141 PMC8870502

[4] Moss, B. & Smith, G. L. in *Fields Virology*, Vol. 2 (eds. Howley, P. M. & Knipe, D.M.) Ch. 16, 573–613 (Wolters Kluwer, 2021).

[5] Jezek, Z. & Fenner, F. Human monkeypox. *Monogr. Virol.* **17**, 1–140 (1988).

[6] Doty, J. B. et al. Assessing monkeypox virus prevalence in small mammals at the human-animal interface in the Democratic Republic of the Congo. *Viruses* **9**, 283 (2017). 10.3390/v9100283 PMC5691634 28972544

[7] Likos, A. M. et al. A tale of two clades: monkeypox viruses. *J. Gen. Virol.* **86**, 2661–2672 (2005). 16186219 10.1099/vir.0.81215-0

[8] Happi, C. et al. Urgent need for a non-discriminatory and non-stigmatizing nomenclature for monkeypox virus. *PLoS Biol.* **20**, e3001769 (2022). 35998195 10.1371/journal.pbio.3001769 PMC9451062

[9] Forni, D., Molteni, C., Cagliani, R. & Sironi, M. Geographic structuring and divergence time frame of monkeypox virus in the endemic region. *J. Infect. Dis.* **227**, 742–751 (2023). 35831941 10.1093/infdis/jiac298 PMC10044091

[10] Heymann, D. L., Szczeniowski, M. & Esteves, K. Re-emergence of monkeypox in Africa: a review of the past six years. *Br. Med. Bull.* **54**, 693–702 (1998). 10326294 10.1093/oxfordjournals.bmb.a011720

[11] Nolen, L. D. et al. Extended human-to-human transmission during a monkeypox outbreak in the Democratic Republic of the Congo. *Emerg. Infect. Dis.* **22**, 1014–1021 (2016). 27191380 10.3201/eid2206.150579 PMC4880088

[12] Vakaniaki, E. H. et al. Sustained human outbreak of a new MPXV clade I lineage in eastern Democratic Republic of the Congo. *Nat. Med* **30**, 2791–2795 (2024). 38871006 10.1038/s41591-024-03130-3 PMC11485229

[13] Yinka-Ogunleye, A. et al. Outbreak of human monkeypox in Nigeria in 2017-18: a clinical and epidemiological report. *Lancet Infect. Dis.* **19**, 872–879 (2019). 31285143 10.1016/S1473-3099(19)30294-4 PMC9628943

[14] Gigante, C. M. et al. Multiple lineages of monkeypox virus detected in the United States, 2021-2022. *Science* **378**, 560–564 (2022). 36264825 10.1126/science.add4153 PMC10258808

[15] Moss, B. Smallpox vaccines: targets of protective immunity. *Immunol. Rev.* **239**, 8–26 (2011). 21198662 10.1111/j.1600-065X.2010.00975.x PMC3074351

[16] Rao, A. K. et al. Use of JYNNEOS (smallpox and monkeypox vaccine, live, nonreplicating) for preexposure vaccination of persons at risk for occupational exposure to orthopoxviruses: recommendations of the Advisory Committee on Immunization Practices-United States, 2022. *Mmwr-Morbidity Mortal. Wkly. Rep.* **71**, 734–742 (2022). 10.15585/mmwr.mm7122e1 PMC9169520 35653347

[17] Deputy, N. P. et al. Vaccine effectiveness of JYNNEOS against Mpox disease in the United States. *New Engl. J. Med.* **388**, 2434–2443 (2023). 10.1056/NEJMoa2215201 PMC10962869 37199451

[18] Dalton, A. F. et al. Estimated effectiveness of JYNNEOS vaccine in preventing Mpox: a multijurisdictional case-control study—United States, August 19, 2022–March 31, 2023. *MMWR Morb. Mortal. Wkly Rep.* **72**, 553–558 (2023). 37200229 10.15585/mmwr.mm7220a3 PMC10205167

[19] Rosenberg, E. S. et al. Effectiveness of JYNNEOS vaccine against diagnosed Mpox infection—New York, 2022. *MMWR Morb. Mortal. Wkly Rep.* **72**, 559–563 (2023). 37339074 10.15585/mmwr.mm7220a4 PMC10205166

[20] Zaeck, L. M. et al. Low levels of monkeypox virus-neutralizing antibodies after MVA-BN vaccination in healthy individuals. *Nat. Med.* **29**, 270–278 (2023). 10.1038/s41591-022-02090-w PMC9873555 36257333

[21] Collier, A.Y. et al. Decline of Mpox Antibody Responses After Modified Vaccinia Ankara-Bavarian Nordic Vaccination. *JAMA* **332**, 1669–1672 (2024). 10.1001/jama.2024.20951 PMC11581614 39361499

[22] Leeuwen, L. et al. Orthopoxvirus-specific antibodies wane to undetectable levels 1 year after MVA-BN vaccination of at-risk individuals, the Netherlands, 2022 to 2023. *Eurosurveillance* **29**, 8–12. 10.2807/1560-7917.Es.2024.29.38.2400575 (2024). 10.2807/1560-7917.ES.2024.29.38.2400575 PMC11484288 39301741

[23] Oom, A. L. et al. The two-dose MVA-BN mpox vaccine induces a nondurable and low avidity MPXV-specific antibody response. *J. Virol*. 10.1128/jvi.00253-25 (2025). 10.1128/jvi.00253-25 PMC12456018 40162783

[24] Freyn, A. W. et al. An mpox virus mRNA-lipid nanoparticle vaccine confers protection against lethal orthopoxviral challenge. *Sci. Transl. Med.* **15**, eadg3540 (2023). 37792954 10.1126/scitranslmed.adg3540

[25] Hou, F. et al. mRNA vaccines encoding fusion proteins of monkeypox virus antigens protect mice from vaccinia virus challenge. *Nat. Commun.* **14**, 5925 (2023). 37739969 10.1038/s41467-023-41628-5 PMC10516993

[26] Fang, Z. et al. Polyvalent mRNA vaccination elicited potent immune response to monkeypox virus surface antigens. *Cell Res.* **33**, 407–410 (2023). 36879038 10.1038/s41422-023-00792-5 PMC9988199

[27] Sang, Y. et al. Monkeypox virus quadrivalent mRNA vaccine induces immune response and protects against vaccinia virus. *Signal Transduct. Target. Therapy*. 10.1038/s41392-023-01432-5 (2023). 10.1038/s41392-023-01432-5 PMC10144886 37117161

[28] Yang, X. D. et al. Evaluation and comparison of immune responses induced by two Mpox mRNA vaccine candidates in mice. *J. Med. Virol.* **95**, e29140 (2023). 10.1002/jmv.29140 37800627

[29] Zuiani, A. et al. A multivalent mRNA monkeypox virus vaccine (BNT166) protects mice and macaques from orthopoxvirus disease. *Cell* **187**, 1363–1373.e1312 (2024). 38366591 10.1016/j.cell.2024.01.017

[30] Mucker, E. M. et al. Comparison of protection against mpox following mRNA or modified vaccinia Ankara vaccination in nonhuman primates. *Cell* **187**, 5540–5553.e10 (2024). 10.1016/j.cell.2024.08.043 39236707

[31] Valenzuela, P., Medina, A., Rutter, W. J., Ammerer, G. & Hall, B. D. Synthesis and assembly of hepatitis B virus surface antigen particles in yeast. *Nature* **298**, 347–350 (1982). 7045698 10.1038/298347a0

[32] Kirnbauer, R., Booy, F., Cheng, N., Lowy, D. R. & Schiller, J. T. Papillomavirus L1 major capsid protein self-assembles into virus-like particles that are highly immunogenic. *Proc. Natl. Acad. Sci. USA* **89**, 12180–12184 (1992). 1334560 10.1073/pnas.89.24.12180 PMC50722

[33] Li, T. C. et al. Expression and self-assembly of empty virus-like particles of hepatitis E virus. *J. Virol.* **71**, 7207–7213 (1997). 9311793 10.1128/jvi.71.10.7207-7213.1997 PMC192060

[34] Nguyen, B. & Tolia, N. H. Protein-based antigen presentation platforms for nanoparticle vaccines. *NPJ Vaccines* **6**, 70 (2021). 33986287 10.1038/s41541-021-00330-7 PMC8119681

[35] Hills, R. A. & Howarth, M. Virus-like particles against infectious disease and cancer: guidance for the nano-architect. *Curr. Opin. Biotechnol.* **73**, 346–354 (2022). 34735984 10.1016/j.copbio.2021.09.012 PMC8555979

[36] Zakeri, B. et al. Peptide tag forming a rapid covalent bond to a protein, through engineering a bacterial adhesin. *Proc. Natl. Acad. Sci. USA* **109**, E690–E697 (2012). 22366317 10.1073/pnas.1115485109 PMC3311370

[37] Thrane, S. et al. Bacterial superglue enables easy development of efficient virus-like particle based vaccines. *J. Nanobiotechnol.* **14**, 30 (2016). 10.1186/s12951-016-0181-1 PMC4847360 27117585

[38] Brune, K. D. et al. Plug-and-display: decoration of virus-Like particles via isopeptide bonds for modular immunization. *Sci. Rep.* **6**, 19234 (2016). 26781591 10.1038/srep19234 PMC4725971

[39] Janitzek, C. M. et al. Bacterial superglue generates a full-length circumsporozoite protein virus-like particle vaccine capable of inducing high and durable antibody responses. *Malar. J.* **15**, 545 (2016). 27825348 10.1186/s12936-016-1574-1 PMC5101663

[40] Cohen, A. A. et al. Construction, characterization, and immunization of nanoparticles that display a diverse array of influenza HA trimers. *PLoS One* **16**, e0247963 (2021). 33661993 10.1371/journal.pone.0247963 PMC7932532

[41] Lampinen, V. et al. SpyTag/SpyCatcher display of influenza M2e peptide on norovirus-like particle provides stronger immunization than direct genetic fusion. *Front Cell Infect. Microbiol.* **13**, 1216364 (2023). 37424789 10.3389/fcimb.2023.1216364 PMC10323135

[42] Heinimaki, S. et al. Antigenicity and immunogenicity of HA2 and M2e influenza virus antigens conjugated to norovirus-like, VP1 capsid-based particles by the SpyTag/SpyCatcher technology. *Virology* **566**, 89–97 (2022). 34894525 10.1016/j.virol.2021.12.001

[43] Keech, C. et al. Phase 1-2 trial of a SARS-CoV-2 recombinant spike protein nanoparticle vaccine. *N. Engl. J. Med.* **383**, 2320–2332 (2020). 10.1056/NEJMoa2026920 PMC7494251 32877576

[44] Tan, T. K. et al. A COVID-19 vaccine candidate using SpyCatcher multimerization of the SARS-CoV-2 spike protein receptor-binding domain induces potent neutralising antibody responses. *Nat. Commun.* **12**, 542 (2021). 33483491 10.1038/s41467-020-20654-7 PMC7822889

[45] Geng, Q. et al. Novel virus-like nanoparticle vaccine effectively protects animal model from SARS-CoV-2 infection. *PLoS Pathog.* **17**, e1009897 (2021). 34492082 10.1371/journal.ppat.1009897 PMC8448314

[46] Zhang, B. et al. A platform incorporating trimeric antigens into self-assembling nanoparticles reveals SARS-CoV-2-spike nanoparticles to elicit substantially higher neutralizing responses than spike alone. *Sci. Rep.* **10**, 18149 (2020). 33097791 10.1038/s41598-020-74949-2 PMC7584627

[47] Cohen, A. A. et al. Mosaic RBD nanoparticles protect against challenge by diverse sarbecoviruses in animal models. *Science* **377**, eabq0839 (2022). 35857620 10.1126/science.abq0839 PMC9273039

[48] Fogg, C. et al. Protective immunity to vaccinia virus induced by vaccination with multiple recombinant outer membrane proteins of intracellular and extracellular virions. *J. Virol.* **78**, 10230–10237 (2004). 15367588 10.1128/JVI.78.19.10230-10237.2004 PMC516428

[49] Rahikainen, R. et al. Overcoming symmetry mismatch in vaccine nanoassembly through spontaneous amidation. *Angew. Chem. Weinh. Bergstr. Ger.* **133**, 325–334 (2021). 10.1002/ange.202009663 PMC10947127 38504824

[50] Williamson, J. D., Reith, R. W., Jeffrey, L. J., Arrand, J. R. & Mackett, M. Biological characterization of recombinant vaccinia viruses in mice infected by the respiratory route. *J. Gen. Virol.* **71**, 2761–2767 (1990). 2254756 10.1099/0022-1317-71-11-2761

[51] Frey, S. E. et al. Human antibody responses following vaccinia immunization using protein microarrays and correlation with cell-mediated immunity and antibody-dependent cellular cytotoxicity responses. *J. Infect. Dis.* **224**, 1372–1382 (2021). 33675226 10.1093/infdis/jiab111 PMC8861366

[52] Earl, P. L., Americo, J. L. & Moss, B. Development and use of a vaccinia virus neutralization assay based on flow cytometric detection of green fluorescent protein. *J. Virol.* **77**, 10684–10688 (2003). 12970455 10.1128/JVI.77.19.10684-10688.2003 PMC228521

[53] Americo, J. L., Moss, B. & Earl, P. L. Identification of wild-derived inbred mouse strains highly susceptible to monkeypox virus infection for use as small animal models. *J. Virol.* **84**, 8172–8180 (2010). 20519404 10.1128/JVI.00621-10 PMC2916512

[54] Americo, J. L., Earl, P. L. & Moss, B. Virulence differences of mpox (monkeypox) virus clades I, IIa, and IIb.1 in a small animal model. *Proc. Natl. Acad. Sci. USA* **120**, e2220415120 (2023). 36787354 10.1073/pnas.2220415120 PMC9974501

[55] Americo, J. L. et al. Susceptibility of the wild-derived inbred CAST/Ei mouse to infection by orthopoxviruses analyzed by live bioluminescence imaging. *Virology* **449**, 120–132 (2014). 24418545 10.1016/j.virol.2013.11.017 PMC3902144

[56] Earl, P. L., Americo, J. L., Cotter, C. A. & Moss, B. Comparative live bioluminescence imaging of monkeypox virus dissemination in a wild-derived inbred mouse (Mus musculus castaneus) and outbred African dormouse (*Graphiurus**kelleni*). *Virology* **475**, 150–158 (2015). 25462355 10.1016/j.virol.2014.11.015 PMC4280325

[57] Ignowski, J. M. & Schaffer, D. V. Kinetic analysis and modeling of firefly luciferase as a quantitative reporter gene in live mammalian cells. *Biotechnol. Bioeng.* **86**, 827–834 (2004). 15162459 10.1002/bit.20059

[58] Luker, K. E. & Luker, G. D. Applications of bioluminescence imaging to antiviral research and therapy: multiple luciferase enzymes and quantitation. *Antivir. Res* **78**, 179–187 (2008). 18358543 10.1016/j.antiviral.2008.01.158 PMC2430099

[59] Kibungu, E. M. et al. Clade I-associated Mpox cases associated with sexual contact, the Democratic Republic of the Congo. *Emerg. Infect. Dis.* **30**, 172–176 (2024). 38019211 10.3201/eid3001.231164 PMC10756366

[60] Kenner, J., Cameron, F., Empig, C., Jobes, D. V. & Gurwith, M. LC16m8: an attenuated smallpox vaccine. *Vaccine* **24**, 7009–7022 (2006). 17052815 10.1016/j.vaccine.2006.03.087 PMC7115618

[61] Grabenstein, J. D. & Hacker, A. Vaccines against mpox: MVA-BN and LC16m8. *Expert Rev. Vaccines* **23**, 796–811 (2024). 39188013 10.1080/14760584.2024.2397006

[62] Boulter, E. A. Protection against poxviruses. *Proc. R. Soc. Med.* **62**, 295–297 (1969). 4307230 10.1177/003591576906200349 PMC1815349

[63] Turner, G. S. & Squires, E. J. Inactivated smallpox vaccine: immunogenicity of inactivated intracellular and extracellular vaccinia virus. *J. Gen. Virol.* **13**, 19–25 (1971). 5130569 10.1099/0022-1317-13-1-19

[64] Earl, P. L., Americo, J. L. & Moss, B. Natural killer cells expanded in vivo or ex vivo with IL-15 overcomes the inherent susceptibility of CAST mice to lethal infection with orthopoxviruses. *PLoS Pathog.* **16**, e1008505 (2020). 32320436 10.1371/journal.ppat.1008505 PMC7197867

[65] Thornhill, J. P. et al. Monkeypox virus infection in humans across 16 countries-April–June 2022. *N. Engl. J. Med.* **387**, 679–691 (2022). 35866746 10.1056/NEJMoa2207323

[66] Dekkers, G. et al. Affinity of human IgG subclasses to mouse Fc gamma receptors. *MAbs* **9**, 767–773 (2017). 28463043 10.1080/19420862.2017.1323159 PMC5524164

[67] Graham, V. A. et al. Efficacy of primate humoral passive transfer in a murine model of pneumonic plague is mouse strain-dependent. *J. Immunol. Res.* **2014**, 807564 (2014). 25097863 10.1155/2014/807564 PMC4109106

[68] Muthumani, K. et al. In vivo protection against ZIKV infection and pathogenesis through passive antibody transfer and active immunisation with a prMEnv DNA vaccine. *NPJ Vaccines* **1**, 16021 (2016). 29263859 10.1038/npjvaccines.2016.21 PMC5707885

[69] Howard, M. K. et al. H5N1 whole-virus vaccine induces neutralizing antibodies in humans which are protective in a mouse passive transfer model. *PLoS One* **6**, e23791 (2011). 21876771 10.1371/journal.pone.0023791 PMC3158096

[70] Ortiz, A. M. et al. Butyrate administration is not sufficient to improve immune reconstitution in antiretroviral-treated SIV-infected macaques. *Sci. Rep.* **12**, 7491 (2022). 35523797 10.1038/s41598-022-11122-x PMC9076870

[71] Townsley, A. C. & Moss, B. Two distinct low-pH steps promote entry of vaccinia virus. *J. Virol.* **81**, 8613–8620 (2007). 17553884 10.1128/JVI.00606-07 PMC1951335

[72] Wyatt, L. S., Earl, P. L., Eller, L. A. & Moss, B. Highly attenuated smallpox vaccine protects mice with and without immune deficiencies against pathogenic vaccinia virus challenge. *Proc. Natl. Acad. Sci. USA* **101**, 4590–4595 (2004). 15070762 10.1073/pnas.0401165101 PMC384791

[73] Blasco, R. & Moss, B. Role of cell-associated enveloped vaccinia virus in cell-to-cell spread. *J. Virol.* **66**, 4170–4179 (1992). 1602540 10.1128/jvi.66.7.4170-4179.1992 PMC241220

[74] Lustig, S. et al. Combinations of polyclonal or monoclonal antibodies to proteins of the outer membranes of the two infectious forms of vaccinia virus protect mice against a lethal respiratory challenge. *J. Virol.* **79**, 13454–13462 (2005). 16227266 10.1128/JVI.79.21.13454-13462.2005 PMC1262616

